# P-1951. Utilization of Intensive Care Interventions in Critically Ill Patients with Candidemia vs. Bacteremia: Implications for Empiric Antifungal Prescribing

**DOI:** 10.1093/ofid/ofaf695.2119

**Published:** 2026-01-11

**Authors:** Mary North Jones, Masayuki Nigo, Stefano Casarin, James Kurian, Aarjav Sanghvi, David Enshuo, Stephen Jones, Ashton Connor, David B Corry, Cesar A Arias, Max W Adelman

**Affiliations:** Baylor College of Medicine, Houston, Texas; Houston Methodist Hospital, Houston, Texas; Houston Methodist Hospital, Houston, Texas; Houston Methodist, Houston, Texas; Houston Methodist Hospital, Houston, Texas; Houston Methodist Hospital, Houston, Texas; Houston Methodist Hospital, Houston, Texas; Houston Methodist Hospital, Houston, Texas; Baylor College of Medicine, Houston, Texas; Houston Methodist and Weill Cornell Medical College, Houston, TX; Houston Methodist Hospital, Houston, Texas

## Abstract

**Background:**

*Candida* spp. are among the most common cause of intensive care unit (ICU)-onset bloodstream infection (BSI), with associated mortality ranging from 30-60%. Yet, there is no strong guidance on when to initiate empiric antifungals for ICU patients suspected of BSI. We compared patients with ICU-onset candidemia vs ICU-onset bacteremia to determine which patients may benefit from the addition of empiric antifungals to standard antibiotic therapy.

**Figure 1:**
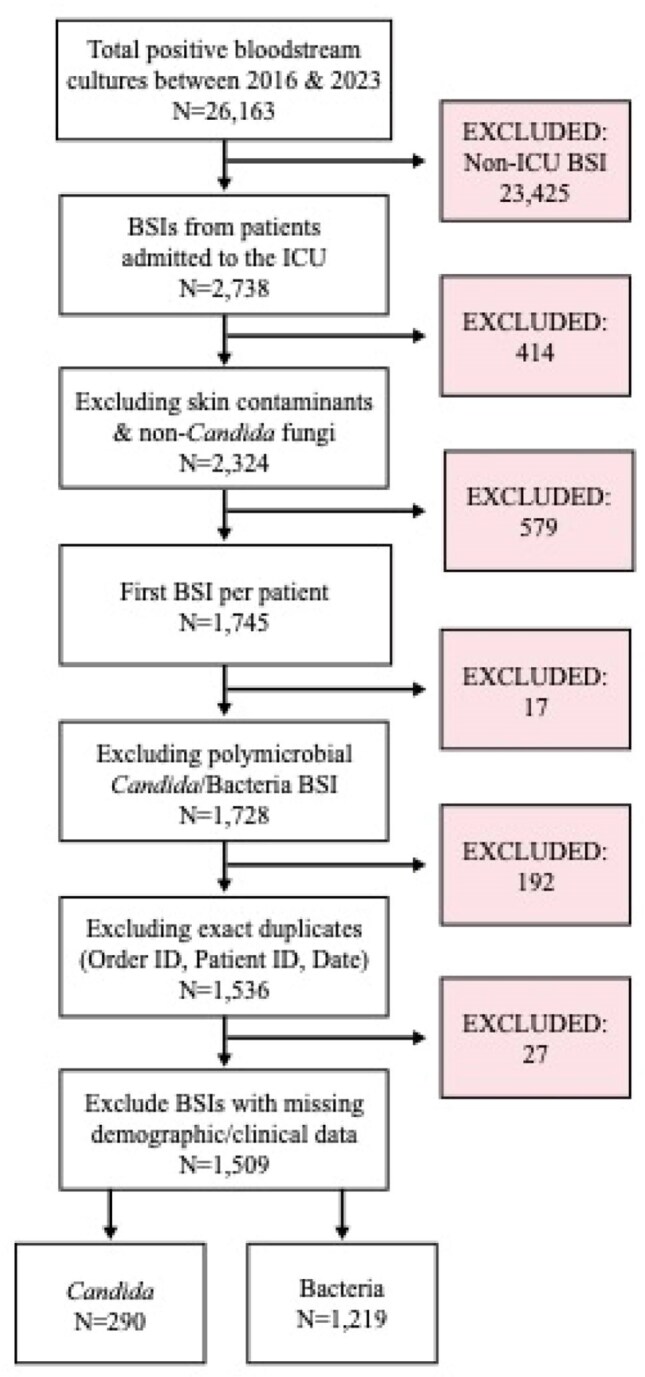
Total number of bloodstream infections (BSIs) collected 2016-2023, inclusion and exclusion criteria, and ultimate number of intensive care unit (ICU) BSIs included in analysis, primary cohort.

**Figure 2:**
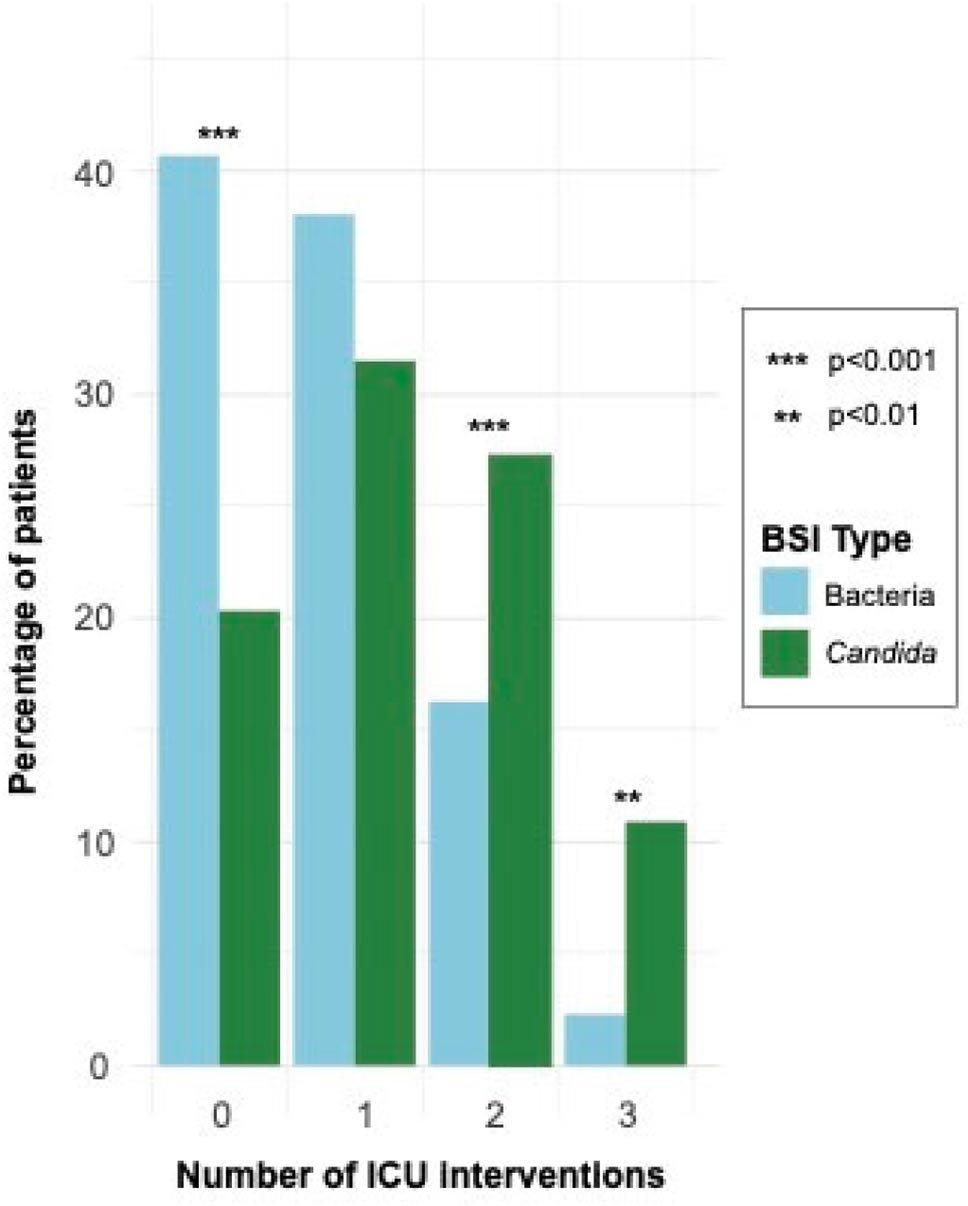
Distribution of intensive care unit (ICU) interventions grouped by bloodstream infection (BSI) type, primary cohort. ICU interventions include invasive mechanical ventilation, administration or one or more vasopressor, and continuous renal replacement therapy.

**Methods:**

We included ICU patients from a hospital system in Houston, Texas with a BSI from 2016-2023 and validated our findings using ICU patients with a BSI in the publicly available MIMIC-IV cohort. We compared characteristics between patients with bacteremia vs candidemia at time of culture. Additionally, we determined risk factors for death and constructed a multivariable regression to determine if candidemia is an independent risk factor for death.

**Figure 3:**
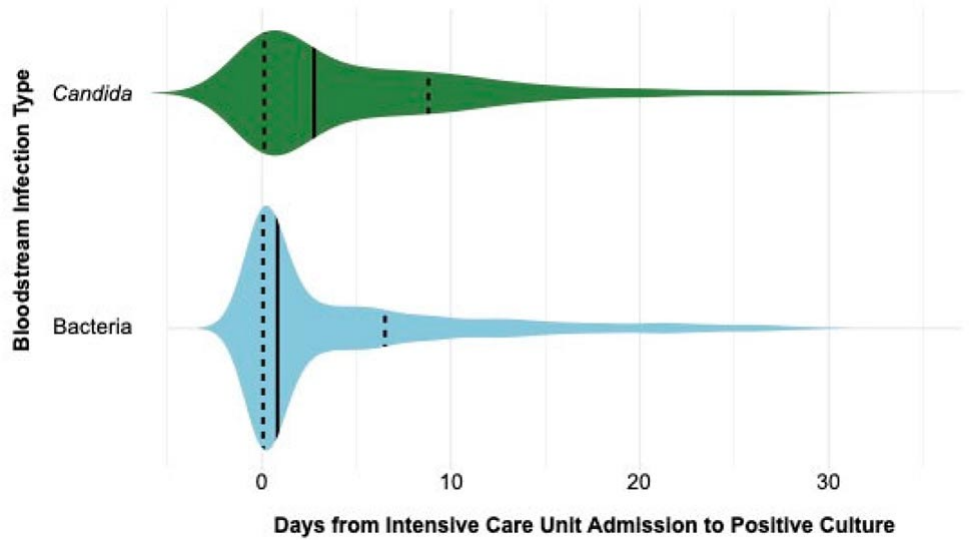
Violin plot depicting days from intensive care unit (ICU) admission to culture, primary cohort. ICU admission = day 0.

**Figure 4:**
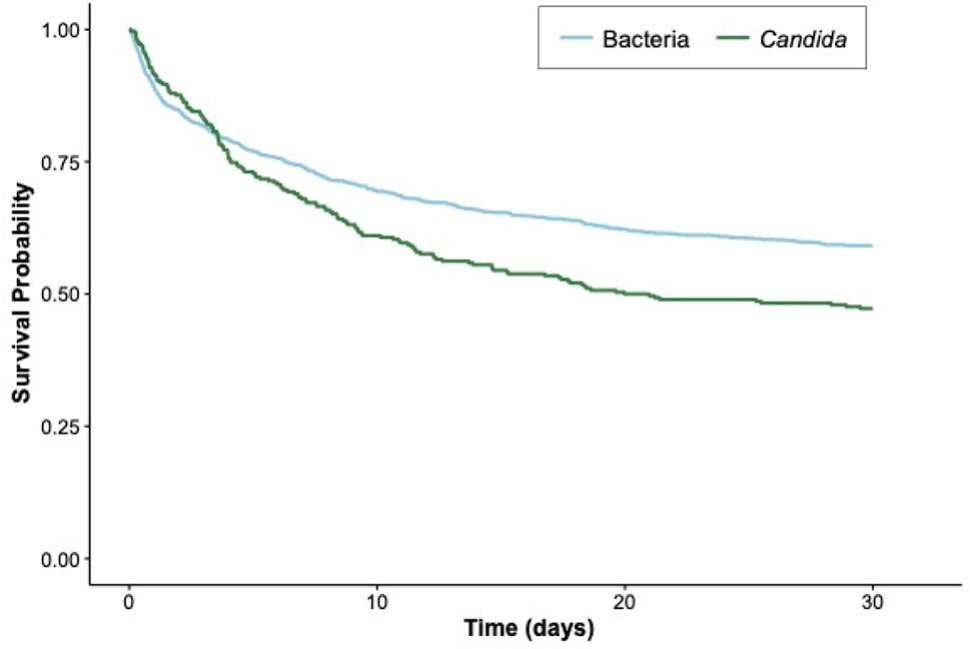
Kaplan-Meier survival curve of 30-day mortality for candidemia and bacteremia patients, primary cohort. Day 0 represents the date of positive culture.

**Results:**

Of 1,509 total patients in our primary cohort, 290 (19.2%) had candidemia and 1,219 (80.8%) had bacteremia [Figure 1].Patients with candidemia were more likely to be on invasive mechanical ventilation (72.8% vs 52.8%, p< 0.001), vasopressors (39.3% vs 24.0%, p< 0.001), and continuous renal replacement therapy (CRRT) (15.2% vs 9.4%, p=0.006) at the time of culture [Figure 2]. They contracted infection later in ICU stay than bacteremic patients (2.8 days vs 0.8 days, p< 0.001) [Figure 3] and were more likely to die within 30 days of culture (unadjusted OR 1.62, 95% CI 1.25-2.09) [Figure 4]. After adjusting for ICU interventions (invasive mechanical ventilation, vasopressors, and CRRT) and baseline parameters, candidemia was not independently associated with mortality compared to bacteremia in our primary cohort (OR 1.21, 95% CI 0.92-1.60) but was in the MIMIC-IV cohort (OR 1.48, 95% CI 1.00-2.17).

**Conclusion:**

We found that the sickest patients in the ICU—those receiving mechanical ventilation, vasopressors, and CRRT—are at a markedly higher risk of contracting candidemia. Candidemia was independently associated with mortality in the MIMIC-IV cohort. Our study highlights the need for further research to clarify the role of empiric antifungal therapy in high-ICU utilizers with suspected BSI.

**Disclosures:**

All Authors: No reported disclosures

